# Clinical Recollections

**Published:** 1896-05-16

**Authors:** Benjamin Ward Richardson


### MEDICAL PROGRESS AND HOSPITAL CLINICS.

[*The Editor will be glad to receive offers of co-operation and contributions from members of the profession. All letters should be addressed to* THE EDITOR, AT THE OFFICE, 428, STRAND, LONDON, W.C.]

### CLINICAL RECOLLECTIONS.

By Sir BENJAMIN WARD RICHARDSON, M.D., F.R.S.

### III.—The Alcoholic Habit.

Compared with the *ad captandum* method of prescribing wine, beer, whiskey, or other alcoholic drink because the patient is low, or assumes to be low, the method of prescribing alcohol itself in diluted doses is very much more precise and more likely to be effective than any other plan. The disadvantage of the measured plan is that so soon as the patient forms the idea that alcohol is being given, he does not generally understand the reason why it is given in the specific manner named. If he may take alcohol, he may as well take it in the wine which he has got stowed away, and the strength of which he assumes to know, as in any other form; but worse remains. If he continues to take even the measured dose as prescribed, he is never satisfied with it as he obtained it originally. He is never clear that the dose given him is sufficient, and so he requests that it may be increased in strength, or he believes it necessary to take it more frequently than it was ordered to be taken, which is the same thing, until at last he is actually taking a prescribed dose in dangerous quantities. We must not wonder at this effect, for it is a special property of alcohol to assert itself. A dose of an ounce or an ounce and a-half which will do for to-day will not do for to-day month, and so it is possible for the most conscientious physician to create an alcoholic temperament and even an alcoholic habit. I do not think I ever did that myself, because I would never sanction an increase of quantity, or too frequently repeat a dose, but the resolution has led to distrust, and has caused patients to seek other advice more pliant and more compatible than mine with their own sensations; and here lies the danger of dealing with alcohol at all as a food or as a sustaining substance for a wearied body. In plain language alcohol is not in any sense a food; it does not contain the great element which builds up the tissues, but it stimulates the moving and secreting organs to greater action, a dangerous expedient in all cases, and in every case nothing but an expedient. The body is not built on a chapter of expedients; the heart is constructed and functioned to give forth a certain number of contractions and relaxations during its life, and, in all probability, every other part of the body is in like manner apportioned for its duties. If, therefore, we give a substance which unduly interferes with the life balance we do evil. We may make the heart beat more quickly for a short time; we may make the glands secrete more freely for a short time, but it is only for a short time, and any overwork we enforce is merely an excess in a limited period which ought to be diffused, or spread out, over a much longer period. So in the end, though we may relieve passing symptoms, we do not lengthen, but really shorten, living function. Moreover, to those of us who have fought the battle carefully, it is quite clear, both in health and disease, that that body works best which undergoes no over-stimulation. I am as convinced of this as I am of anything in the way of phenomenon that has appeared to me. Some of the most industrious persons I have ever met with; some who have had the most varied talents and capabilities; some who have laboured for the longest periods, have done all gracefully—and usually easily—without any assistance from stimulation.

If we tell a man to take a sharp walk in an ascent, or to run on a level ground, or to enter into a heated argument; for the moment that he is obeying us we are administering a stimulant just as certainly as if we were giving him brandy. We may be advising what may appear as a brilliant effort, but it is an effort bought at a cost that is ruinous to the man who displays it, and, on the whole, we are not adding, but taking away from the life value of the person who is under our command. Meanwhile we are acting on what is sometimes called a practice, but what is really a false principle, on which we cannot hold our own in the present days of enlightened criticism. It is a common argument amongst those with whom we come in contact that if they can do without alcohol they are certainly better without it in any form. The admission is quite new, and I well remember the time when it would never have been made except by the extremist abstainer for abstinence's sake. Now it is nearly universal as an admission.

"I go," said a patient to me the day I write this—"I go frequently for five or six weeks at a time and never touch a drop of alcohol, and it is quite fair to say that during this abstinence I do everything better than at any other time. I eat better, I think better, I work better, I sleep better, nor do I know any reason why I should change from that order of things, for a day or two in the country, or a short voyage would be quite sufficient to recruit me if I did not feel well. There comes, however, a moment when I meet with old boon companions; there is a dinner, or supper, a laugh or a joke, and I am led to join with the rest in what is called conviviality. Then I feel next day the qualms that have arisen. I have been comparatively moderate, but next morning breakfast is not so agreeable as usual; there is some derangement about the digestive system, so that the bowels are uneasy, or even relaxed; I pass more urine; I am troubled with cold; I crave for a repetition of what has brought about these symptoms; I feel drowsy; I certainly do not collect my facts and figures as usual, and I go to bed to pass a more restless night than is ordinarily the case. It takes me three or four days to come out of this struggle into which I never ought to have entered."

This is the common story of a young, or even a middle-aged man. It increases in intensity as the years go on, if the habit be not entirely ignored, and it must be said, with much regret, that, instead of leading to the ignoring of habit as would be most desired, it sometimes intensifies the habit, and from a temporary change in the organic functions leads ultimately to what may be called a permanent irregularity—to an actual disease, in which the liver and kidney, and, almost necessarily with them, the heart, undergoes a permanent change of an unfavourable character.

